# Carnosine retards tumor growth in vivo in an NIH3T3-HER2/neu mouse model

**DOI:** 10.1186/1476-4598-9-2

**Published:** 2010-01-06

**Authors:** Christof Renner, Nadine Zemitzsch, Beate Fuchs, Kathrin D Geiger, Matthias Hermes, Jan Hengstler, Rolf Gebhardt, Jürgen Meixensberger, Frank Gaunitz

**Affiliations:** 1 Klinik und Poliklinik für Neurochirurgie, Universitätsklinikum Leipzig und Medizinische Fakultät der Universität Leipzig, 04103 Leipzig, Germany; 2 Institut für Medizinische Physik und Biophysik, Universitätsklinikum Leipzig und Medizinische Fakultät der Universität Leipzig, 04103 Leipzig, Germany; 3 Institut für Pathologie, Technische Universität Dresden, 01307 Dresden, Germany; 4 Leipniz-Institut für Arbeitsforschung, TU Dortmund, 44139 Dortmund, Germany; 5 Institut für Biochemie, Universitätsklinikum Leipzig und Medizinische Fakultät der Universität Leipzig, 04103 Leipzig, Germany

## Abstract

**Background:**

It was previously demonstrated that the dipeptide carnosine inhibits growth of cultured cells isolated from patients with malignant glioma. In the present work we investigated whether carnosine also affects tumor growth in vivo and may therefore be considered for human cancer therapy.

**Results:**

A mouse model was used to investigate whether tumor growth in vivo can be inhibited by carnosine. Therefore, NIH3T3 fibroblasts, conditionally expressing the human epidermal growth factor receptor 2 (HER2/neu), were implanted into the dorsal skin of nude mice, and tumor growth in treated animals was compared to control mice. In two independent experiments nude mice that received tumor cells received a daily intra peritoneal injection of 500 μl of 1 M carnosine solution. Measurable tumors were detected 12 days after injection. Aggressive tumor growth in control animals, that received a daily intra peritoneal injection of NaCl solution started at day 16 whereas aggressive growth in mice treated with carnosine was delayed, starting around day 19. A significant effect of carnosine on tumor growth was observed up to day 24. Although carnosine was not able to completely prevent tumor growth, a microscopic examination of tumors revealed that those from carnosine treated animals had a significant lower number of mitosis (p < 0.0003) than untreated animals, confirming that carnosine affects proliferation in vivo.

**Conclusion:**

As a naturally occurring substance with a high potential to inhibit growth of malignant cells in vivo, carnosine should be considered as a potential anti-cancer drug. Further experiments should be performed in order to understand how carnosine acts at the molecular level.

## Background

Carnosine, a dipeptide discovered more than 100 years ago [1] is a naturally occurring substance synthesized by endogenous carnosine synthetase. It is present in mammalian brain at a concentration between 0.7 and 2.0 mM [2] and reaches concentrations of up to 20 mM in skeletal muscle [3]. However, not much is known about its physiological function but several possible roles have been considered since its first discovery (for detailed review see [4,5]). Among these functions ph-buffering [6], metal chelation [7] or neurotransmitter function [8] have been discussed and the currently most intensively debated aspects are its potential protective effect against oxidative stress [9], its likely role as a therapeutic agent for the treatment of Alzheimer's disease [10] and its use as a potential anti-senescence drug [11]. Just recently, it was demonstrated that carnosine is also able to inhibit growth of cultured cells isolated from human brain tumors in cell culture. This effect is not accompanied by apoptotis or necrosis, but is rather caused by reduced proliferation [12]. In fact, more recently published data indicates that reduced proliferation is mediated by an influence on glycolytic energy metabolism [13]. These interesting results indicate that carnosine may have an anti tumorigenic effect. In order to ask, whether carnosine might also be able to inhibit growth of tumors in vivo, we were looking for an experimental model in order to test a potential therapeutic applicability of carnosine. We decided to employ an approved animal model. This model uses murine NIH3T3 fibroblasts conditionally expressing human HER2/neu under the control of a tetracycline-responsive promoter, that allows turning off of expression under the influence of tetracycline or its derivatives [14]. HER2/neu, also termed neu or erbB-2, belongs to the epidermal growth factor receptors, involved in cell cycle control and cell differentiation. Its activation induces several signal transduction pathways including the ras/MAP kinase pathway [15,16] and the phosphatidyl inositol 3,4,5-triphosphate kinase/AKT pathway [17]. In the experiments performed, fibroblasts expressing the HER2/neu receptor were subcutaneously injected into the dorsal skin of nude mice, resulting in the growth of solid tumors. Two series of experiments were performed with animals, that were treated daily with and without carnosine after the subcutaneous injection of HER2/neu expressing fibroblasts. For several weeks, tumor size of animals, receiving carnosine or NaCl as control, was determined daily.

## Methods

### Chemicals and reagents

If not stated otherwise all chemicals were purchased from Sigma (Taufkirchen, Germany).

### Cell culture

The tetracycline-controlled cell line NIH3T3-HER2/neu [14] was cultivated in 75-cm^2 ^flasks (TPP, Trasading, Germany) in DMEM-Medium (high glucose, Invitrogen, Karlsruhe, Germany) supplemented, with 10% fetal bovine serum (FCS Gold; PAA, Cölbe, Germany) and antibiotics (50 μg/ml streptomycin and 30 μg/ml penicillin) at 37°C and 5% CO_2 _in humidified air. Harvest of cells was performed using accutase (PAA) after cells reached 90% confluency.

### Animals

Female nude mice Crl:CD^®^-1-Foxn1^nu ^were purchased from Charles River Laboratories (Sulzfeld, Germany). The animals had access to standard chow (Altrumin pellets; Altrumin GmbH, Lage, Germany) and received water ad libitum.

### Cell based assays

For the determination of ATP concentration and dehydrogenase activity, cells were cultivated in 96 well plates (black, clear bottom; μClear, Greiner Bio One, Frickenhausen, Germany) in 200 μl of medium. After 4 hours, medium was exchanged and fresh medium containing carnosine was added. After 70 hours, intracellular adenosine triphosphate (ATP) concentration was measured by the CellTiter-Glo Assay (Promega, Mannheim, Germany) as described [18]. Briefly, after incubation in the absence or presence of carnosine, medium was removed and exchanged for 50 μl of fresh medium without carnosine. At this point it should be noted, that removal of medium is not necessary and does not change the results. We included this step in order to make the experiments absolutely comparable to previously published experiments [12]. Then, 50 μl of CellTiter-Glo reagent were added. Ten minutes after addition of reagent and incubation at room temperature, luminescence was determined using a SpectraMax M5 Multilabel Reader (Molecular Devices, Ismaning, Germany) in luminescence mode with an integration time of 500 ms.

For the determination of dehydrogenase activities the CellTiter-Blue assay (Promega) was employed. Briefly, before the start of the assay, cells received 100 μl of fresh medium without carnosine and 20 μl of CellTiter-Blue reagent. Then, cells were incubated for 90 min at 37°C, 5% CO_2 _and in humidified air in an incubator. Finally, fluorescence was determined using an M5 Multilabel Reader with an excitation wave length of 560 nm and an emission wave length of 590 nm.

### Analysis of mitotic activity

Tumors were excised and fixed in formalin for at least 24 hrs. Then the tissues were dehydrated and embedded in paraffin wax. Five μm thick serial sections of whole tissue were cut with a rotation microtome (Leica, Germany), deparaffinized and stained with Hematoxylin-Eosin (Merck, Germany). The number of mitoses was counted at 40 × magnification on a Zeiss Axioplan 2- microscope (Carl Zeiss, Oberkochen, Germany) with a graded ocular lens (10 × 10 squares, corresponding to 250 × 250 μm at 40 × magnification). All areas of the tissues were counted (maximum 10 High Power fields (HPF)) and the rate of mitosis was calculated as mean per one High-Power field. Statistical analysis used the students t-test.

### Determination of carnosine concentrations in serum

Serum samples were applied onto a MALDI target as 1 μl droplets. The matrix 2,5-dihydroxybenzoic acid (DHB) was added as 1 μl droplet (0.5 mol/l stock solution in methanol) containing pentaglycine (Gly-Gly-Gly-Gly-Gly) as 5 mmol/l reference standard onto the sample. Positive ion MALDI-TOF mass spectra were acquired on a Bruker Daltonics Autoflex workstation (Bruker, Germany). The system utilizes a pulsed nitrogen laser emitting at 337 nm and spectra were recorded in the reflector mode under "delayed extraction" conditions. The extraction voltage was 20 kV and 128 single laser shots were averaged for each mass spectrum. The sum of the intensities of the H^+^, Na^+ ^and K^+ ^adducts of carnosine (*m/z *= 227, 249 and 265) and of pentaglycine (*m/z *= 304, 326 and 342) were determined for quantitative analysis using the software "Flex Analysis" version 2.2 (Bruker Daltonics, Germany).

### Statistics

All data are expressed as Mean ± standard deviation. Analysis of significance was performed using the two sample t-test implemented in the Origin 7.5 Software (OriginLab Corporation, Northampton, USA).

## Results

### Carnosine inhibits growth of NIH3T3-HER2/neu cells in culture

**Figure 1:**
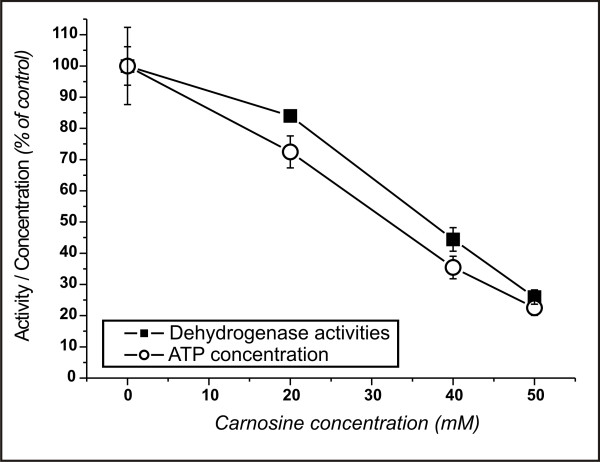
**Activity of dehydrogenases and concentration of ATP in NIH3T3-HER2/neu cells cultivated at different concentrations of carnosine**. Cells were incubated for 70 hours in media with carnosine. After incubation ATP concentration as well as dehydrogenase activities were determined and compared to cells not treated with carnosine.

Before the experiments with animals were initiated, it was asked, whether NIH3T3-HER2/neu cells are influenced by carnosine in culture, as demonstrated for cells from human glioblastoma [12]. Therefore, NIH3T3-HER2/neu cells were cultivated at an initial density of 500 cells per well in 96-well plates. Four hours after the start of the culture, carnosine was added at different concentrations, and cells were cultivated for 70 hours. Carnosine was added from a 1 M stock solution in 0.7% NaCl with pH adjusted to that of cell culture medium (ph = 7.4). Besides, we showed that addition of carnosine to culture medium does not influence pH under the culture conditions (37°C and 5% CO_2 _[12]). Control cells received a medium containing the corresponding amount of 0.7% NaCl solution. After the incubation time, the concentration of ATP was measured and dehydrogenase activity was determined. As can be seen in Fig. 1, carnosine reduces dehydrogenase activity by ~20% at 20 mM, by ~60% at 40 mM and by ~80% at 50 mM carnosine. The concentration of ATP is reduced by ~30% at 20 mM, by ~70% at 40 mM and by ~80% at 50 mM carnosine. This result is in good agreement with the previously published data from tumor cells derived from patients with malignant glioma and it encouraged to ask whether the effect of carnosine on tumor cell growth may also be observable in vivo.

### Intraperitoneal delivered carnosine is detectable in the blood

In order to answer the question whether intraperitoneal delivered carnosine will enter the circulation, 3 animals received an intraperitoneal injection of 500 μl carnosine (1 M) dissolved in NaCl-Solution (0.7%), ph 7.4. One μl samples of blood from the tails were analysed for the presence of carnosine in serum by MALDI TOF mass spectrometry. The concentrations determined after injection were 20 ± 5 mM after 15 min, 32 ± 4.2 mM after 30 min, 10 ± 0 mM after 60 min and 15 ± 0 mM after two hours. In mice, not treated with carnosine, the mean concentration determined was 0.097 ± 0,031 mM and therefore already below the limit of quantification (0.2 mM) but above the limit of detection (0.05 mM).

### Tumor growth in the NIH3T3-HER2/neu animal model under the influence of carnosine

**Figure 2:**
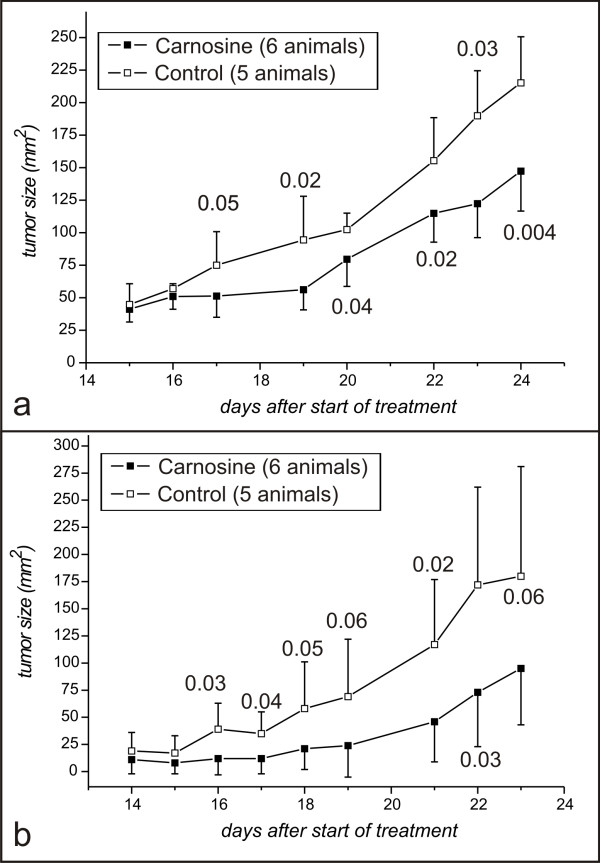
**Development of NIH3T3-HER2/neu tumors in animals treated with carnosine**. The size of tumors developed from subcutaneous implanted NIH3T3-HER2/neu cells was determined by measuring maximum and minimum diameters. The tumor size presented is the product of these two diameters measured at different days after the start of the experiment. Part a and part b of the figure represent two independent series of experiments. The numbers in the figure indicate the level of significance as determined by student's t-test (p < 'number indicated').

In two independent experiments (each with 11 animals) 7 × 10^6 ^NIH3T3-HER2/neu cells in 100 μl sterile phosphate-buffered saline (PBS: 2.7 mM KCl, 1.5 mM KH_2_PO_4_, 140 mM NaCl, 6.5 mM Na_2_HPO_4_, pH 7.4) were injected subcutaneous into the dorsal skin of female nude mice. The eleven animals in each of the two series were divided into two groups. One group (6 animals) received a daily intraperitoneal injection of 500 μl carnosine (1 M), dissolved in NaCl-Solution (0.7%), ph 7.4. The second group (5 animals) was used as control and received a daily injection of 500 μl of 0.9% NaCl, pH 7.4. All injections were performed for six days with a one day break, starting again for six days. Measurable tumors were detected 12 days after injection, and tumor size was determined by measuring the maximum and minimum diameters with a caliber rule. The product of these two diameters was defined as the tumor size with the dimension mm^2^. The result of the two experiments is presented in Fig. 2. As can be seen, progressive tumor development starts at day 16 in control mice and at day 19 in carnosine mice. Hypothesis testing using students t-test indicated significances between treated and control animals from day 17 to day 24 in the first series and from day 17 to day 23, as indicated by the numbers in Fig. 2. Statistical analysis was not performed after 24 days, since at this time some animals already reached a tumor size in the control population, that was not bearable with animal care. When the tumors reached the critical size (>220 mm^2^), the animals were treated with anhydrotetracycline (animals from first series; Fig. 2a) or were sacrificed for microscopic analysis of tumors (animals from second series; Fig. 2b).

### Development of tumors after switching off HER2/neu expression

In the first experimental series (Fig. 2a) the animals were not sacrificed but were further treated. The treatment protocol and the detailed tumor progression is presented as additional material. Briefly, the animals received subcutaneous injections of 100 μl anhydrotetracycline (12 mg/ml) every second day, after tumors reached a critical size (>220 mm^2^). These injections turn off HER2/neu expression and result in the disappearance of tumors. In previous experiments, a reoccurrence of tumors was observed after 20 to 45 days after anhydrotetracycline administration [14]. Since these new tumors were reported do be independent from HER2/neu expression, we wondered whether carnosine may also have an effect on the reoccurrence of tumors. In our experiments, all tumors treated with anhydrotetracycline disappeared but no recurrence of tumors was detected. In addition, one mouse had a spontaneously diminishing tumor. This animal never received anhydrotetracycline, but was continuously treated with carnosine, until a second tumor developed, that reached a critical size around day 60. At this time anhydrotetracycline was injected followed by a significant decrease of tumor size, indicating that this tumour was still resulting from the injected NIH3T3-HER2/neu cells.

### Mitotic activity is reduced in tumors from animals treated with carnosine

**Figure 3:**
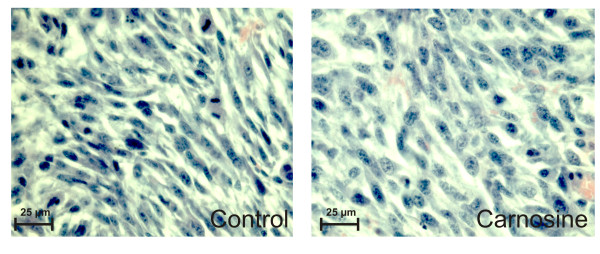
**Microscopic images from tumors stained with Hematoxylin-Eosin from an animal treated with carnosine (right) and from a control animal treated with NaCl solution (left)**. Many mitotic figures are seen in the tumor from the non-treated animal compared to the tumor from the treated animal. Pictures were taken at an original magnification of 400×.

In the second series of experiments (Fig. 2b) the animals were not treated with anhydrotetracycline. Instead, the animals were sacrificed and the tumours removed in order to analyse mitotic activity. Paraffin imbedded sections were analysed as described in the Materials and Methods section. Microscopy revealed that all tumors showed a highly malignant mostly sarcoma-like morphology. Compared to the tumours from non-treated animals, the nuclei of carnosine treated tumours were less pleomorph (Fig. 3). Most interestingly, the tumours in the carnosine treated group exhibited a significantly lower number of mitoses per high power field (14.0 ± 1.58 mitoses/HPF) compared to the tumors from control mice (21.6 ± 2.19 mitoses/HPF; P < 0.0003).

## Discussion

The search for potent anticancer drugs is still a huge challenge. The two most challenging aspects are on one hand to find drugs with minor side effects and on the other hand to find substances that are able to prevent growth of tumors that can not be successfully treated yet. Carnosine is a highly interesting candidate with regard to both aspects. Firstly, carnosine is a naturally occurring substance that is present in muscle and other organs. Secondly, experiments with cultured cells from non-curable malignant glioma demonstrated that carnosine is able to diminish proliferation of these cells. Just recently, it was demonstrated that carnosine inhibits glycolysis and ATP synthesis in tumor cells in culture [13]. This observation is in contrast to the well-known fact that in normal cells carnosine improves both processes [19]. The basic difference with regard to energy metabolism between tumor cells and normal cells have been described almost a century ago and are known as the Warburg effect [20]. The molecular mechanisms of this effect are still not completely understood. In fact, the study of the mechanisms, on how carnosine affects normal and tumor cell energy metabolism differentially, may help to exploit the basic differences, and may suggest a way for the creation of a knowledge-based strategy to fight cancer without affecting normal cells [21]. However, and first of all, it had to be asked whether carnosine can be administered as a drug. In the present study, we therefore investigated whether carnosine may in fact prevent growth of tumor cells in an established mouse model based on NIH3T3 cells expressing the oncogene HER2/neu [14,22]. HER2/neu belongs to the human epidermal growth factor receptor family (HER) of tyrosine kinases. It was first described to be overexpressed in 25-30% of breast cancers, but its overexpression is also seen in subsets of gastric, esophagal and endometric cancers and in some cancers of the oropharynx, the lung and the bladder (for review see [23]). Just rececently, it was demonstrated that HER2 is also expressed in glioblastoma patient samples [24]. In addition, Herceptin, a recombinant humanised anti-HER2/neu antibody, can induce cell death in cell lines derived from glioblastomas [25]. In the mouse model employed in the present study, expression of HER2/neu is strongly required for tumor growth. Switching off expression by the administration of anhydrotetracycline results in rapid tumor regression. Therefore, it is tempting to speculate that carnosine also affects HER2/neu expression, but recent data more strongly indicates that carnosine exhibits its effect at the level of glycolysis, depleting ATP production, finally resulting in reduced proliferation [13]. In the present work the anti proliferative effect of carnosine in vivo is demonstrated by the reduced growth of tumors in treated animals and by the smaller number of mitotic cells in treated tumors. One interesting challenge is now, how to enhance the local concentration of carnosine in hope to get a stronger effect on tumor growth. In fact, the carnosine concentration in the serum of treated animals after ~26 days of treatment and 24 hours after the last injection is not significantly different from the carnosine concentration in the serum of animals not treated with carnosine (data not shown). We also do not know whether the continuous administration of carnosine may induce carnosinase, the degrading enzyme, that in humans is known to be secreted from the liver into the serum [26]. Of course, an intratumoral injection of carnosine might have been a possibility but this would have made the determination of tumor size in the experiments presented difficult. Considering a clinical application of carnosine, an intratumoral injection of carnosine or the implantation of wafers (e.g. into the resection cavity of a surgically excised tumor in the case of malignant gliomas), that continuously release carnosine, may be discussed. However, discussing a potential clinical application, we should not withhold, that two minor tumors developed in two carnosine treated mice of the first series and one minor tumor in two carnosine treated mice of the second series, close to the time, when the primary tumor already had reached its maximum size (data not shown). The additional tumors were very small and regressed after administration of anhydrotetracycline. Although they may have been initiated in the vicinity of the primary tumor by a small number of cells subcutaneously introduced by the injection, it cannot be ruled out, that they originated from cells that have migrated from the primary tumor. Whether carnosine may induce a migratory potential, is pure speculative and needs further experimental data.

One puzzling observation in the course of the experiments was the complete disappearance of one tumor in a mouse, that was continuously treated with carnosine. A careful examination of the mouse did not reveal any sign of an accidental injection that is usually detectable even after several days. In addition, regression needs several repeated injections and cannot just be caused by one accidental injection. Therefore, it must be concluded that the tumor regressed under the continuous treatment with carnosine. Although this tumor reappeared after 50 days of treatment, this observation is a highly interesting one with regard to a possible use of carnosine in human tumor therapy.

## Conclusions

The data presented prove that carnosine has an antiproliferative effect on malignant cells in vivo as demonstrated before in culture. Therefore, carnosine should be considered as a potential anticancer agent, especially since it is a naturally occurring substance. It is now very important to analyse how carnosine inhibits proliferation and whether it may be a useful drug for anticancer therapy in humans.

## Competing interests

The authors declare that they have no competing interests.

## Authors' contributions

CR and NZ carried out the animal experiments and the cell based assays. BF carried out the MALDI experiments. KG carried out the microscopic study and analysed mitotic activity. MH and JH advised the animal experiments and supplied know how as well as the NIH3T3-Her2/neu cells. RG and JM helped to draft the manuscript. FG designed and coordinated the study, performed animal experiments and cell based assays, performed statistical analysis and drafted the pictures and the manuscript. All authors read and approved the manuscript.
